# Cardiac effects of anabolic steroid use amongst recreational body builders - a CMR study

**DOI:** 10.1186/1532-429X-14-S1-P186

**Published:** 2012-02-01

**Authors:** Tevfik F Ismail, Peter J Angell, Andrew Jabbour, Gillian Smith, Rick Wage, Benjamin Hewins, Niraj Mistry, Annette L Dahl, Susan Clark, Bethan Cowley, Keith George, Gregory Whyte, Dudley J Pennell, Sanjay K Prasad

**Affiliations:** 1 CMR Unit & NHLI Imperial College London, Royal Brompton Hospital & NHLI Imperial College London, London, UK; 2 Research Institute for Sport and Exercise Sciences, Liverpool John Moores University, Liverpool, UK

## Background

Abuse of anabolic steroids to induce skeletal muscle hypertrophy is widespread amongst recreational bodybuilders, however, the cardiac effects of such drugs have not been systematically documented. The ability of cardiovascular magnetic resonance (CMR) to image the myocardium in any plane and achieve full myocardial coverage renders it the gold standard for assessing LV volumes and mass.We sought to investigate the cardiac effects of anabolic steroid use with CMR hypothesising that significant hypertrophy and distinct LV remodelling would be seen in steroid users relative to non-users.

## Methods

A total of 23 recreational bodybuilders were studied - 15 anabolic steroid users and 8 non-users. CMR was undertaken on a 1.5T Avanto (Siemens, Erlangen, Germany) using breath-hold steady-state free precession cine sequences. Three long-axis images were obtained followed by sequential short axis cines from the AV-groove to the apex. Late gadolinium enhancement (LGE) imaging was performed ~10 min after intravenous Gadovist 0.1 mmol/kg (Schering, Berlin, Germany). Indexed LV and RV ventricular volumes, ejection fraction and indexed LV mass were determined using a semi-automated threshold-based algorithm after manual tracing of epicardial and endocardial borders (CMRtools, London, UK). LV remodelling index was calculated as the ratio of LV-mass to LV end-diastolic volume (LV-EDV).

## Results

None of the subjects exhibited any late gadolinium enhancement. Mean indexed LV and RV volumes and EF were comparable for the two groups (Table 1). However, there was significant left ventricular hypertrophy (LVH) in the steroid users with both increased indexed LV mass and peak wall thickness relative to non-users (Table 1). Peak wall thickness often exceeded the >15 mm cut off used to diagnose hypertrophic cardiomyopathy (range 10-17 mm in steroid users versus 7-11 mm in non-users). The LV remodelling index was significantly higher in the steroid users versus the controls (Table 1) implying increases in LV mass with relatively preserved volumes in comparison to the more eccentric LV remodelling seen in the non-users.

**Table 1:** 

	Anabolic Steroid Users (mean±SD, n=15)	Non-users (mean±SD, n=8)	P Value
**Age (years)**	30.1±4.92	28.8±6.15	0.61
**Body Surface Area (m^2^)**	2.15±0.21	1.99±0.15	0.077
**Indexed LV-EDV (g/m^2^)**	92.8±12.2	94.1±12.1	0.82
**Indexed LV-ESV (g/m^2^)**	36.4±6.55	35.3±5.98	0.70
**LV EF (%)**	61.1±2.66	62.5±3.33	0.27
**Indexed LV Mass (g/m^2^)**	96.3±13.9	82.4±15.0	0.037
**Maximum Wall Thickness (mm)**	12.9±2.2	9.38±1.3	<0.001
**LV Remodeling Index (g/ml)**	1.05±0.16	0.88±0.11	0.015
**Indexed RV-EDV (g/m^2^)**	95.3±29.6	100±18.2	0.69
**Indexed RV-ESV (g/m^2^)**	36.4±6.55	35.3±5.98	0.70
**RV EF (%)**	51.9±3.9	59.0±4.7	0.003

## Conclusions

Anabolic steroid use amongst bodybuilders can induce significant left ventricular hypertrophy. The pattern and severity of remodelling can mimic hypertrophic cardiomyopathy. The use of such drugs should be considered in the differential diagnosis of significant otherwise unexplained left ventricular hypertrophy, particularly in the athletic cohort.

## Funding

This work is supported by the NIHR Cardiovascular Biomedical Research Unit at the Royal Brompton and Harefield NHS Foundation Trust, and Imperial College. Dr Ismail is supported by the British Heart Foundation.

